# The Increased Level of Serum p53 in Hepatitis B-Associated Liver Cirrhosis

**Published:** 2014-09

**Authors:** Parisa Shahnazari, Kourosh Sayehmiri, Zarrin Minuchehr, Ardavan Parhizkar, Hossein Poustchi, Ghodratollah Montazeri, Ashraf Mohamadkhani

**Affiliations:** 1 Monoclonal Antibody Research Centre, Avicenna Research Institute, ACECR, Tehran, Iran;; 2 Psychosocial Injuries Research Centre, Ilam University of Medical sciences, Ilam, Iran;; 3 National Institute of Genetic Engineering and Biotechnology, NIGEB, Tehran, Iran;; 4 Digestive Disease Research Centre, Shariati Hospital, Tehran University of Medical Science, Tehran, Iran

**Keywords:** Chronic hepatitis B, Liver cirrhosis, Hepatitis B virus-encoded X

## Abstract

**Background: **The ability of tumour suppressor protein p53 (P53) to regulate cell cycle processes can be modulated by hepatitis B virus (HBV). While preliminary evidences indicates the involvement of protein-x of HBV (HBx) in altering p53 DNA binding, no further data have been accumulated for the significance of serum p53 in chronic hepatitis B virus infected patients.

**Methods: **72 non-cirrhotic and 19 cirrhotic patients infected by HBV were enrolled for the analysis in this study. Enzyme linked immunosorbent assay (ELISA) was performed to study the concentrations of serum p53 protein. The tertiary structures of HBx and P53 were docked by Z-dock and Hex servers for *in-silico* protein-protein interaction analysis.

**Results: **There was a significant association between the serum p53 and cirrhosis (OR=1.81 95% CI: 1.017-3.2, P=0.044). Cirrhotic patients had higher level of serum p53 compare with chronic infection of HBV (1.98±1.22 vs. 1.29±0.72 U/ml, P=0.05). No evidence of correlation was seen between the different variables such as age, gender, log viral load, serum a*lkaline phosphatase* (ALP) and *alanine aminotransferase* (ALT) with serum p53. Tertiary model shows that the amino acid residues from Arg110 to Lys132 of the N-terminal of P53 which is critical for ubiquitination, are bonded to a region in N- terminal of HBx amino acid residues from Arg19 to Ser33.

**Conclusion: **There is an increase in serum p53 in HBV-related cirrhosis patients. In this case, HBx might be responsible for such higher concentration of p53 through HBx-p53 protein-protein interaction, as is shown by molecular modeling approach.

## Introduction

The infection of hepatitis B virus is one of the most frequent causes of chronic liver disease, though the principal molecular mechanisms responsible for its clinical outcome have not been well characterized.^1^^,^^2^

In response to stress signal, induced by viral components, p53 selectively regulates a set of its target genes to employ its function in tumor suppression.^3^ It is well known that this protein regulates the cell cycle pathways as part of its vital function to keep the genomic stability.^4^^-^^6^ Therefore, p53 prevents the perpetuation of a defective genome and the development of cancer. Remarkably, current studies have focused on the impact of p53 to transactivate genes involved in aerobic metabolism.^4^ Mutations in p53 gene not only considerably drive disease progression, but also have been shown as a valuable tumor marker in most of the tumors.^3^^,^^7^^,^^8^

High prevalence of p53 alterations have been investigated in HCC as a consequence of chronic hepatitis B.^9^^,^^10^ HBV encodes a small protein x (HBx) that seems to play a critical role in hepatocarcinogenesis and found to be expressed in chronic hepatitis, cirrhotic liver, and hepatocellular carcinoma (HCC).^11^^,^^12^ However, preliminary reports verify that the distal C-terminal region of HBx has inhibitory effect on apoptosis through inactivation of p53 in microinjected human fibroblasts.^13^^,^^14^ It has also been proposed that the interaction of HBx with DNA-bound p53 induces a repression domain in HBx leading to inhibition of cellular transcription machinery.^14^

The p53 protein is composed of 393 residues and is divisible into three structural domains. The amino-terminal trans-activation domain, the central ‘core’ domain (residues from 102 to 292) that is essential for sequence-specific DNA binding and the carboxyl terminus oligomerization domain, which is required for the assembly of p53 tetramers and contains nuclear localization signals.^5^ The interaction of HBx protein and p53 tumor suppressor protein has been the subject of examinations in many researches in order to evaluate the biological function of p53 protein in HBV infected cells.^14^^-^^16^

In this study, the level of serum p53 in chronic hepatitis and cirrhotic patients following HBV infection is evaluated. Additionally, the interaction of p53 protein and protein x of HBV is predicted with computational modeling to clarify the excess level of p53 protein in HBV- related cirrhotic patients.

## Patients and Methods

*Patients*

In a cross-sectional study, patients referred to the Hepatitis Clinic of Shariati Hospital during a period of six months were included. All patients were HBsAg positive and HBeAg negative. They were divided into two groups: (a) 72 non-cirrhotic patients with normal or elevated ALT; (b) 19 patients with cirrhosis defined as the presence of ascites and/or oesophageal varices and/or splenomegaly, together with low serum albumin, prolonged prothrombin time and thrombocytopenia at enrolment. Patients with evidence of concomitant hepatitis C or D virus infection, HIV infection, autoimmune, drug-induced or other causes of chronic liver disease were excluded. Serum samples and demographic data were collected at the initial assessment and stored at -70˚C prior to analysis. The protocol of this study was approved by the Ethics Committee of Digestive Disease Research Centre, Tehran University of Medical sciences. 

*Clinical Evaluation and HBV-DNA Quantification*

Serological markers were determined with a commercial ELISA kit (Dia.Pro diagnostic, Milano, Italy). The 91 positive serum samples of HBsAg were evaluated with respect to the presence of p53 protein using the ELISA method (Bender MedSystem, Vienna, Austria). HBV DNA was extracted from 200 ml of serum using the High Pure Viral Nucleic Acid Kit (Roche Germany) and then quantified in the Light-Cycler (Roche) by RealART^TM^ HBV LC PCR (QIAGEN, Hilden, Germany) according to the manufacturer’s instructions. The linear range of this assay is 10^2^–10^9^ copies/ml.

*Hbx* *and p53 Interaction Analysis*

The sequence of protein-x of HBV was obtained by translation of the appropriate reading frame in complete viral genome of HBV genotype D consensus [UniProt: P0C681]. The tertiary structure of Hbx was used from previously gathered data.^17^ The X-ray crystal structure of P53 was retrieved from PDB (2OCJ). The protein-protein interactions were achieved by means of Z-dock (http://zdock.umassmed.edu/)^18^ and HEX (http://hexserver.loria.fr).^19^ KFC2 (http://kfc.mitchell-lab.org)^20^ was used for prediction binding “hot spots” within protein-protein interfaces by recognizing structural features.

*Statistical Analysis*

All statistical analyses were performed with the Statistical Program for Social Sciences (SPSS, version 19 for Windows; SPSS Inc., Chicago, Ill.). The mean±standard deviation (SD) was considered for continuous variables. The associations between variables, age, P53, viral load, ALT and ALP were computed using the Pearson correlation coefficient (r). Normality of variables was checked using One-Sample Kolmogorov-Smirnov Test. The relationship between p53 concentrations and cirrhotic or non-cirrhotic patients was assessed using logistic regression techniques (after control for multiple covariates). In logistic regression models, both Enter and Likelihood forward conditional models were implemented. Statistical significance was defined by a *P* value of less than 0.05.

## Results

*Demographic and Biochemical Characteristics*

From 91 chronic hepatitis B patients with the mean±SD age of 44±13 years, 67 were male (73%) and there was no significant difference in the cases of ALT, HBV DNA and serum p53 among them. The serum levels of p53 showed no significant direct correlations with serum ALT and HBV DNA level. Also no significant correlation was observed between serum P53 and age, though the level of serum ALT had positive correlation with the copies/mL of HBV DNA (r=0.4, P=0.001). 

*Patients’ Clinical Features*

Nineteen patients with the mean age of 54±12 years old (a decade older than patients with chronic hepatitis B (54±12 vs. 42±12 years, P=0.001)) with complication of cirrhosis, showed higher serum p53 (1.98±1.22 vs 1.29±0.72 U/mL; P=0.050). Following age adjustment, the p53 concentrations remained significantly high in cirrhotic patients (P=0.038). Serum p53 had a total mean of 1.32±0.94 U/mL, however in the cirrhotic patients, the number of individuals with the p53 more than 2 U/mL (36.8%) was not significantly greater than the non-cirrhotic patients (27.8%) (P=0.421). The characteristics of subjects in these distinct groups are summarized in table 1.

**Table 1:** Comparable clinical and pathological data of cirrhotic and non-cirrhotic patients

Clinical factor*	**All patients (n=91)**	**Non-cirrhotic (n=72)**	**Cirrhotic (n=19)**	**P value**
Age (Years)	44±13	42±12	54±12	0.001**
log HBV DNA (copies/mL)	3.63±0.94	3.55±0.93	3.92±0.92	0.1
ALT (IU/L)	61±43	60±47	65±30	0.6
ALP (IU/L)	217±80	209±76	246±90	0.6
P53 (U/mL)	1.32±0.94	1.29±0.72	1.98±1.22	0.050**

*Mean±SD; **P value refers to differences between the 5 groups

*Association of Serum p53 Level with Cirrhosis*

To analyze the presence of liver cirrhosis in CHB patients, several characteristics such as p53, age, gender, log viral load and serum ALT and ALP were selected in a multivariable-adjusted logistic regression model. As shown in table 2, there is a significant association between increased serum p53 and cirrhosis (OR=1.81 95% CI: 1.017-3.2, P=0.044). Additionally, no evidence of interactions and multicollinearity between variables (age, gender, log viral load, ALP and ALT) with liver cirrhosis was identified. Moreover, the association of serum level of p53 and age with cirrhosis using conditional likelihood forward method in logistic regression is estimated as:

$$\text{log}\left(\frac{\text{p}}{1-\text{p}}\right)=-\text{5.9}+\text{.58p53}+\text{0.074age}$$

**Table 2:** Two models to predict cirrhosis, in multivariate logistic model age and p53 were associated with liver cirrhosis. Using conditional likelihood forward method in logistic regression as model 2, shows age and p53 are associated with liver cirrhosis independently

**Table 2**		**B**	**S.E.**	**Wald**	**Sig.**	**OR**	**95% CI. for OR**	
							**Lower**	**Upper**
Model 1	Age	0.066	0.025	6.948	0.008	1.068	1.017	1.122
	Sex	-0.105	0.687	0.023	0.879	0.901	0.234	3.465
	ALT	-0.002	0.007	0.101	0.751	0.998	0.983	1.012
	ALP	0.005	0.004	1.400	0.237	1.005	0.997	1.013
	Log_Viral_load	0.391	0.371	1.112	0.292	1.479	0.715	3.061
	p53†	0.593	0.294	4.070	0.044	1.809	1.017	3.219
	Constant	-7.972	2.079	14.701	0.000	0.000		
Model 2	Age	0.074	0.023	10.272	0.001	1.077	1.029	1.126
	p53	0.584	0.281	4.324	0.038	1.793	1.034	3.108
	Constant	-5.939	1.352	19.290	0.000	0.003	Constant	-5.939

CI=Confidence interval; OR=Odds ratio (OR significant [ie, P<0.05] indicated in bold); †Adjusted for the effect of p53 and cirrhosis (OR=1.81 95% CI: 1.017-3.2, P=0.044).

This model shows that p53 and age are two independent predictive factors for the liver cirrhosis.

*In Silico Analysis of p53 Interaction with HBx*

**Figure 1:**
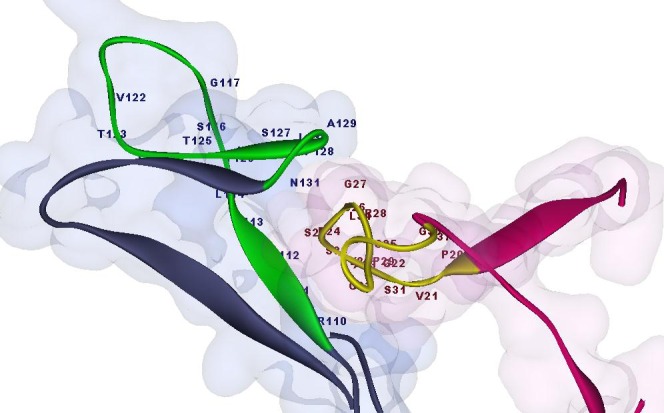
Doking result of P53 and HBx. As shown, the HBx protein (in purple) from Arg19 to Ser33 is bonded to the amino acids Arg110 to Lys132 in N-terminal trans-activation domain (TAD) of P53 protein (in blue).

The model of tertiary structure of HBx was docked with P53 using Z-dock and Hex servers. The best model was selected from the overlapped results and then analyzed with KFC2 server to find hot spots in protein-protein interactions. The data indicated that the residues from Arg110 to Lys132 in the N-terminal trans-activation domain (TAD) of P53 protein bonded to a region in the N- terminal of Hbx protein from Arg19 to Ser33 (figure 1).

KFC2 Server (predicted binding “hot spots” within protein-protein interfaces) showed Arg110, Leu111, Gly112, Phe113, His115, Tyr126, pro128, Ala129 and Asp131 in P53 had high probability to interface with the Hbx protein. The hot spots of Hbx protein are: Arg19, Val21, Ala23, Glu24, Ser25, Cys26, Val30, and Ser33 which was compatible with the present docking data. 

## Discussion

This study describes the serum level of p53 protein in patients with chronic hepatitis B and HBV-related cirrhosis. The findings show that the concentrations of p53 in the serum of patients with HBV-related cirrhosis were significantly higher than its level in patients with chronic hepatitis B. However no significant correlation was noticed between the higher concentration of the p53 protein with age, gender and viral load. Furthermore, the docking results from protein-protein interaction exhibited that the N-terminal of HBx was bonded to the N-terminal trans-activation domain (TAD) in P53. 

The hypothesis that describes the higher p53 level in the serum of HBV-related cirrhotic patients might be the higher expression of HBx in cirrhotic patients as a consequence of viral replication or integration of viral genome in host genome during the end stage of liver disease, and also an increase of p53 protein half-life in its interaction with HBx. To clarify this hypothesis, it is mentioned that Mdm2 (Mouse double minute 2 homolog) which has been recognized as E3 ubiquitin-protein ligase, regulates both the transcriptional activity and the half-life of p53 in a negative feedback loop. This protein targets the N-terminal region of core domain of p53 protein for ubiquitin-mediated proteolysis.^21^ In the other words, P53 turnover is regulated through mono- or poly-ubiquitination^21^^,^^22^ and HBx might interfere with the specific targeting of p53 ubiquitination by Mdm2, therefore, increasing the half-life of p53. Thus the raised levels of serum p53 in cirrhotic patients support the hypothesis of HBx-p53 interaction. Although there is no significant correlation between HBV DNA level and serum p53 in the current findings, but HBx expression from integrated HBV DNA which is common in cirrhotic patients might be responsible for increased level of serum p53 through p53-HBx protein-protein interaction.^15^^,^^21^ In this regard, increased expression of p53 in cirrhotic and HCC has already been reported.^23^

To date, there has been no precise or adequate research showing serum level of p53 in chronic hepatitis B. However, there are some assessments about overexpression or unusual form of p53 protein in the serum.^7^^,^^8^ The unusual form of p53 has been associated with more half-life of this protein and could expose an increase level of this protein in serum.^3^^,^^7^^,^^24^ The results of this study is consistent with a recent study of serum and cytoplasmic p53 concentrations in chronic hepatitis C, in which high level of the serum p53 were detected in the patients with HCC and liver cirrhosis.^23^ In another study, Cheng et al. showed high expression of HBx in all cirrhosis samples by immunohistochemical staining.^25^ There are also some reports on close association between HBV infection and the progressive liver diseases with the abnormalities in p53 tumor suppressor.^4^^,^^15^

Although it is often believed that the p53 is important in responding to DNA damage, oncogenic motivation and hypoxia; increased ability of p53 in regulating some features of cellular metabolism in some disease development is now being revealed.^26^ Hence, the increased level of p53 in patients with advance liver disease underlines the importance of p53 in directing metabolic remodeling and metabolic disorders. This requires further investigation to determine the precise impact of p53 in interaction with viral products on HBV-related cirrhotic patients.

## Conclusion

This pilot study indicates a higher expression of serum p53 in cirrhotic infected patients with HBV. It presents a model for the interaction of p53 and HBx that indicates the role of HBx in cirrhosis patients for increasing p53 half-life. This is a matter for future investigations. Evaluation of p53 protein in serum samples could be a valuable tool for the assessment of HBV-related cirrhosis and chronic hepatitis B patients.
